# Oxidative stress and antioxidant markers in oral leukoplakia: a systematic review and meta-analysis

**DOI:** 10.3389/fmed.2026.1807075

**Published:** 2026-04-21

**Authors:** Xueru Chen, Mengying Shao, Longzhen Liu, Hui Xie, Jincai Guo

**Affiliations:** 1 Department of Pharmacy, Changsha Stomatological Hospital, Changsha, China; 2 School of Stomatology, Hunan University of Chinese Medicine, Changsha, China; 3 Department of Periodontal and Oral Mucosa, Changsha Stomatological Hospital, Changsha, China; 4 Department of Pharmacy, The Second Hospital of Zhuzhou City, Zhuzhou, Hunan, China

**Keywords:** antioxidants, leukoplakia, meta-analysis, oral, oxidative stress

## Abstract

**Objective:**

Oral leukoplakia (OLK) is a common possibly malignant condition of the oral cavity, oxidative stress (OS) are intrinsically linked to the initiation and progression of carcinogenesis. However, previous studies on the changes of various OS and antioxidant markers in patients with OLK and their diagnostic value have been inconsistent, and there is a lack of systematic comparison and evaluation. This study aims to systematically evaluate and compare the status of OS and antioxidant markers in patients with OLK versus healthy controls to identify potential biomarkers for early diagnosis.

**Methods:**

A comprehensive electronic search was conducted across PubMed, Embase, Web of Science, and the Cochrane Library to identify case–control studies investigating antioxidant and OS markers in OLK patients compared to healthy individuals from database inception to May 1, 2025.

**Results:**

Twelve studies were selected for inclusion in this meta-analysis. The analysis focused on five pro-oxidant substances and antioxidant state markers. Our findings revealed that the level of malondialdehyde (MDA) higher in patient with OLK (MD: 0.26, 95% CI: 0.22 to 0.3, *p* < 0.02, I^2^ = 63%), while the levels of key antioxidants, including serum glutathione (GSH, MD: −8.6, 95% CI: −12.97 to −4.23, *p* < 0.00001), superoxide dismutase (SOD, MD: −6.25, 95% CI: −11.45 to −1.05, *p* = 0.02), glutathione peroxidase (GPx, MD: −9.27, 95% CI: −15.72 to −2.82, *p* = 0.005), and catalase (CAT, MD: −25.71, 95% CI: −40.09 to −11.33, *p* = 0.0005), were lower in patient with OLK.

**Conclusion:**

This meta-analysis suggests that OS may be involved in the pathogenesis of OLK. The pooled analysis indicates a potential disruption of redox balance in patients with OLK. These findings provide a basis for further investigation into the mechanisms linking OS to OLK pathogenesis.

**Systematic review registration:**

https://www.crd.york.ac.uk/PROSPERO/#myprospero, CRD42025642682.

## 1 Introduction

Oral leukoplakia (OLK) is a common possibly malignant condition of the oral cavity, characterized by the presence of white patches on the oral mucosa that cannot be removed by scraping (1, 2). Oral cancer is frequently preceded by many oral disorders with malignant potential, with OLK being the most prevalent (3). The occurrence rate of OL is approximately 1.39%, and patients with OLK have a 40.8 times higher risk of developing oral squamous cell carcinoma (OSCC) compared to the general population, with a 5-year transformation rate of approximately 3.3% (4, 5). Additionally, the elevated survival rate and low morbidity linked to OLK emphasize the critical importance of its early detection (6). Currently, OLK is described as “a white patch of uncertain risk after excluding other known diseases or conditions that are not oral cancer”, the diagnosis is excluded primarily, basing on a combination of patient history, clinical assessment, and histopathological findings (7). Consequently, the progress of early detection methods and biomarkers has become progressively essential.

Although the etiology and pathogenesis of OLK remain unclear, research has shown that oxidative stress (OS) as a central driver in its pathogenesis (8). OS is defined as a physiological perturbation caused by an imbalance between the production of reactive oxygen species (ROS) and the biological system’s ability to detoxify these reactive intermediates or repair the resulting damage (9). Under pathological conditions, excessive ROS accumulation overwhelms antioxidant defenses, precipitating inflammatory cascades, lipid peroxidation, and DNA damage—events that are intrinsically linked to the initiation and progression of carcinogenesis. Previous investigations have indicated that patients with OLK manifest significantly comprised antioxidant capacity, evidenced by reduced levels of salivary and serum glutathione S-transferase (GST) and uric acid (UA), alongside elevated markers of lipid peroxidation such as 8-isoprostane (8-ISO) and thiobarbituric acid reactive substances (TBARS) (10).

Therapeutic strategies targeting this redox imbalance have shown promise. Antioxidants, including vitamin E, blackberry extract (BRBE), and proanthocyanidins (PCA), may function as chemopreventive agents by scavenging ROS, enhancing DNA repair mechanisms, and mitigating the cytotoxicity of carcinogens (11). Consequently, quantifying the shift in oxidant/antioxidant balance offers a potential avenue for monitoring disease progression.

In conclusion, a strong correlation exists among OLK, OS, and antioxidants. OS is not only involved in the onset and progression of OLK but also signifies alterations in antioxidant marker levels, showing antioxidant defense capacity and serving as an indicator of potential malignant transformation in OLK. Despite the biological plausibility, a systematic synthesis of evidence quantification assessing OS and antioxidant markers in OLK is currently lacking. Therefore, this study aims to perform a systematic review and meta-analysis to evaluate the alterations of these markers in OLK patients. By clarifying the association between OS, antioxidants and OLK pathophysiology, we hope to provide evidence-based insights to guide future clinical diagnostics and therapeutic management.

## 2 Methods

According to the *Cochrane Handbook* and PRISMA guidelines standard, we have registered with PROSPERO (https://www.crd.york.ac.uk/PROSPERO/#myprospero, CRD42025642682) on May 1st, 2025.

### 2.1 Search strategy

All searches employed a mix of medical subject headings (MeSH terms) and free-text terms based on the PECO framework. A comprehensive search was conducted encompassing four databases—Embase, PubMed, Web of Science and the Cochrane Library ranging from their inception to May 1, 2025. Our focus was on case–control studies that investigated antioxidant and OS markers. Additionally, we also examined the reference lists of the chosen articles to find additional relevant studies.

### 2.2 Eligibility criteria

Studies were included if they met the following criteria based on the PICOS framework:

*Population (P)*: Patients diagnosed with OLK based on clinical and/or histopathological examination.

*Intervention/Exposure (I)*: Measurement of OS or antioxidant markers in serum or saliva.

*Comparison (C)*: Healthy control individuals without OLK or other oral diseases, and without habits of alcohol consumption, betel nut chewing, or smoking.

*Outcomes (O)*: Reported data on OS or antioxidant markers.

*Study design (S)*: Case–control studies, or cross-sectional studies.

The criteria for exclusion: (1) Treated with antioxidant compounds; (2) Without abstract or full-text available; (3) Studies involving patients with multiple oral diseases; (4) incomplete data.

### 2.3 Data collection and quality evaluation

Two independent reviewers (MS, ZL, and XC) screened titles and abstracts, followed by full-text review. Discrepancies were resolved through consultation with senior investigators (JG and HX). Extracted data included: first author, publication year, geographic location, sample size, participant demographics (age/sex), specimen type (serum/saliva), and mean/SD of the markers.

Quality assessment was performed using the Newcastle-Ottawa Scale (NOS). Studies with scores of 7–9 were categorized as high-, 5–6 were categorized as moderate-, and less than 5 points were categorized as low-quality.

### 2.4 Statistical analysis

The sample size and mean ± standard deviation (SD) were used to generate the effect size. Continuous data were analyzed using the mean difference (MD) and 95% confidence interval (CI). Cochran’s *Q* test and the *I*^2^ test (12) were used to evaluate heterogeneity among studies. A fixed-effects model was chosen if no heterogeneity was observed (*I*^2^ ≤ 50%). If statistical heterogeneity was present (*I*^2^ > 50%), further analysis of the source of heterogeneity was performed, after excluding the effects of significant clinical heterogeneity, a random-effects model was used for meta-analysis.

## 3 Results

### 3.1 Literature search

**Figure 1:**
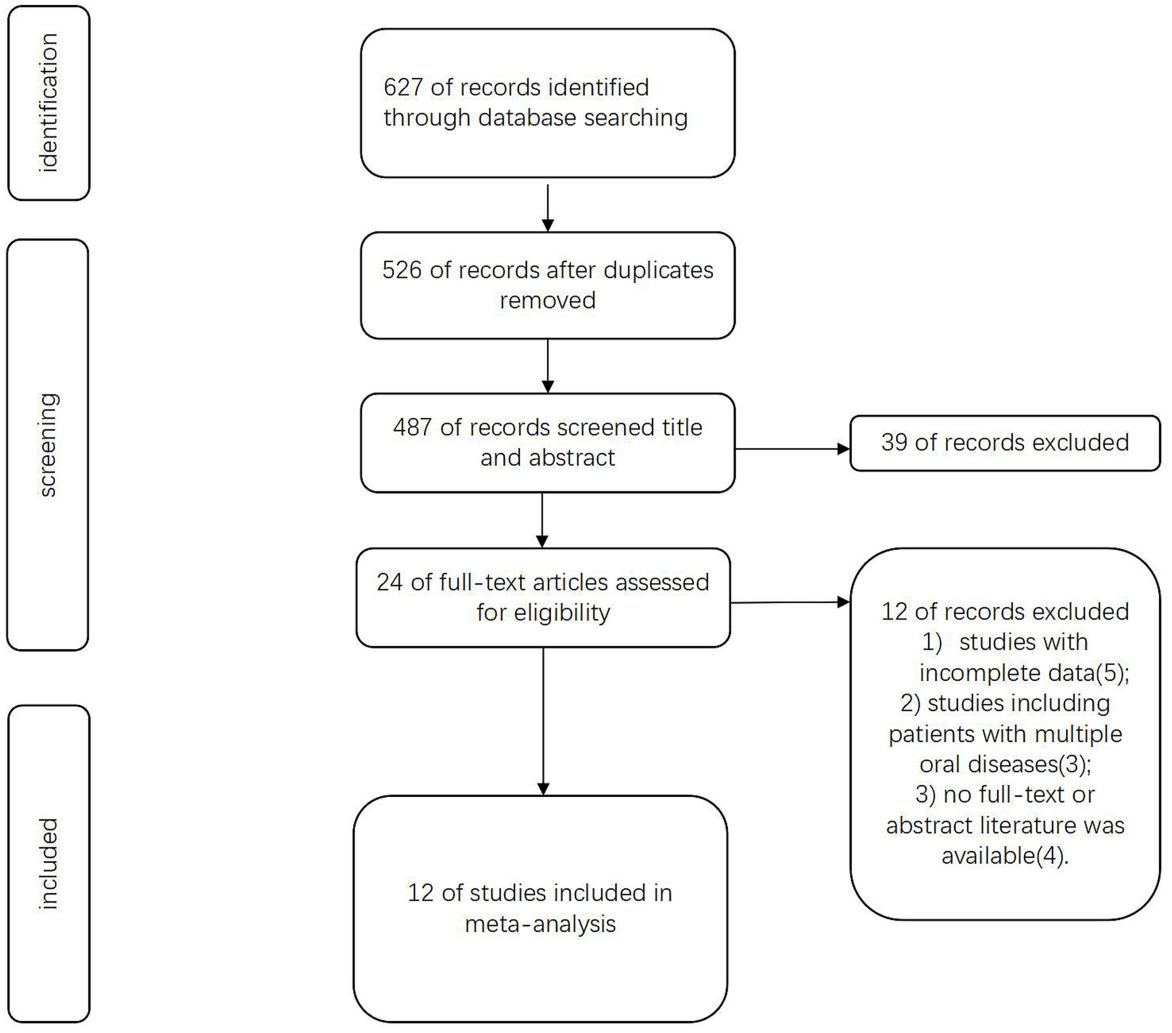
Flowchart of the study.

Figure 1 illustrates the process of study selection. The initial search yielded 627 records. After removing duplicates (n = 101) and screening titles/abstracts, 24 articles remained for full-text assessment. Ultimately, we incorporated 12 publications that fulfilled the requirements for the subsequent meta-analysis.

### 3.2 Study characteristics

**Figure 2:**
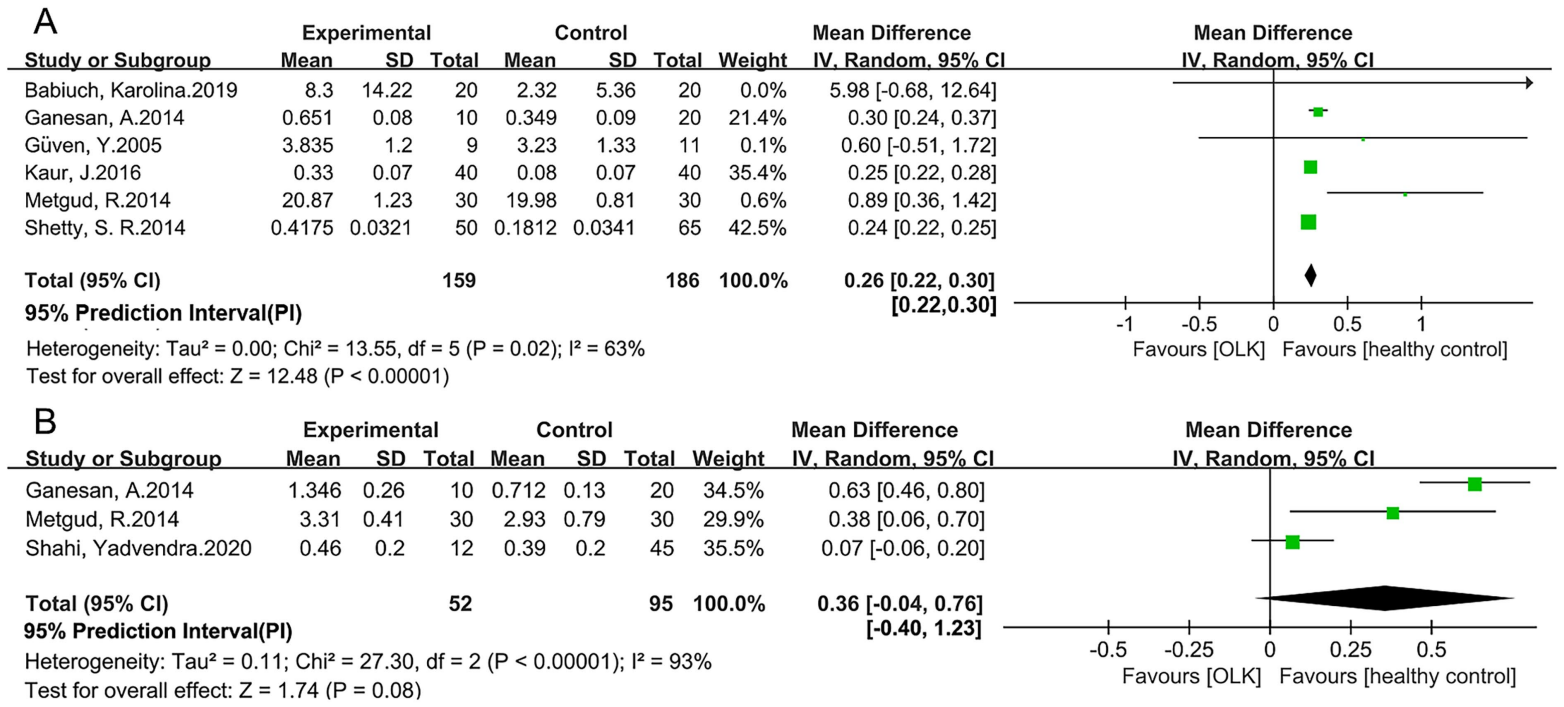
Forest plot for the levels of **(A)** saliva MDA; **(B)** serum MDA in patients with OLK compared with healthy controls.

**Figure 3:**
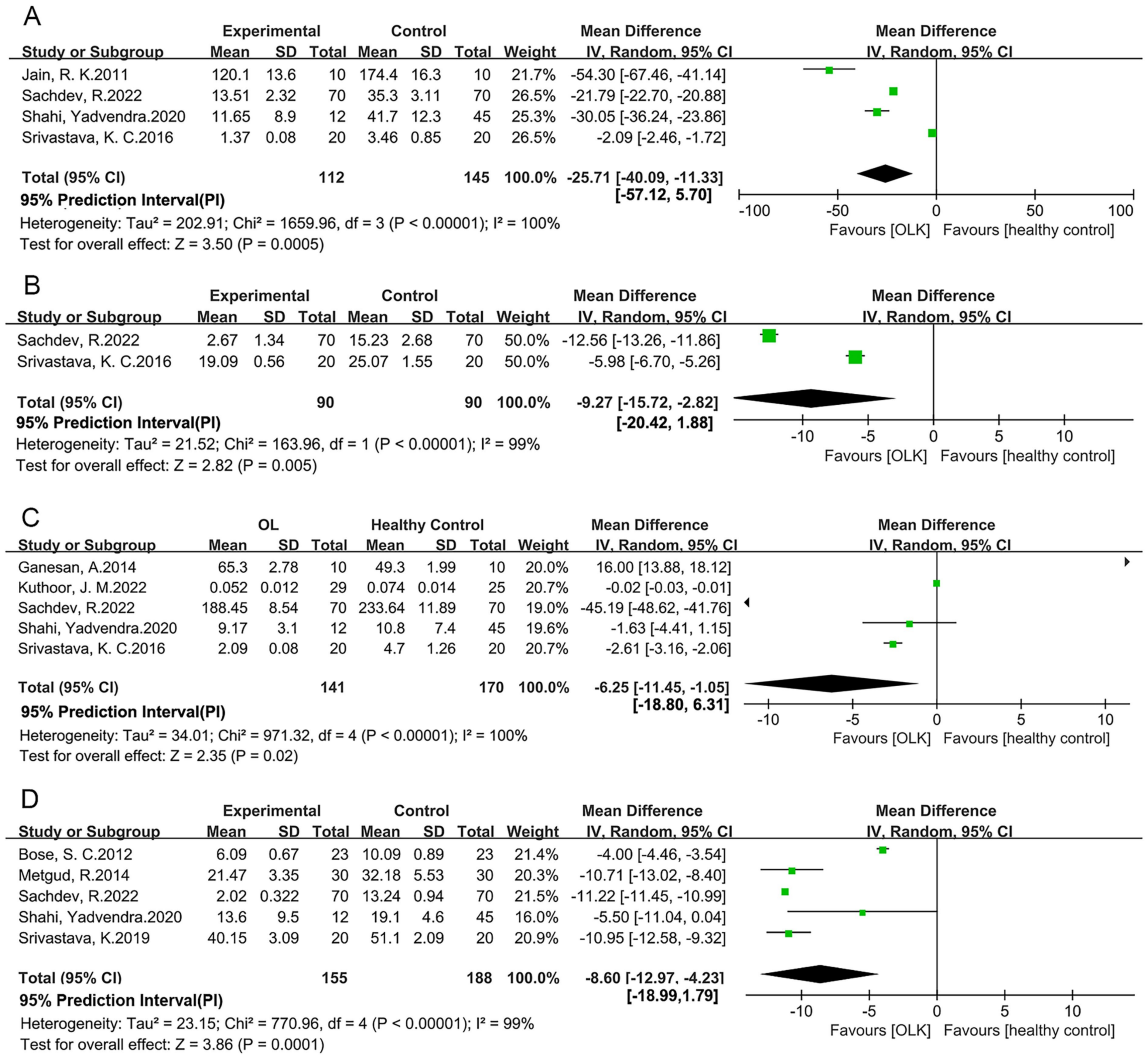
Forest plot for the levels of **(A)** serum CAT; **(B)** serum GPx; **(C)** serum SOD; **(D)** serum GSH in patients with OLK compared with healthy controls.

Table 1 presents a detailed summary of the 12 selected studies, which included 379 healthy controls and 323 patients with OLK. The findings encompassed five antioxidant and OS markers. Figures 2, 3 revealed that there is significant differences in the levels of antioxidant and OS markers between healthy control and patients with OLK. All the studies employed the case–control design. Ten studies were conducted in India, one in Saudi Arabia, and one in Poland. Among the 12 articles analyzed, five studies (10, 13–16) reported serum superoxide dismutase (SOD) levels, two studies (10, 15) reported serum glutathione peroxidase (GPx) levels, four studies (10, 15–17) reported serum catalase (CAT) levels, five studies (10, 15, 16, 18, 19) reported serum glutathione (GSH) levels, three studies (13, 16, 19) reported serum malondialdehyde (MDA) levels, six studies (13, 19–23) reported saliva MDA levels. As shown in Table 2, 12 studies were rated as high quality.

**Table 1:** Characteristics of the studies included in the meta-analysis.

The First author, publication year	Country	Participants		Age (Yr)		Gender (M/F)		Sample
		T	C	T	C	T	C	
Kuthoor et al. (14)	India	29	25	30–70	30–70	—	—	Serum^a^
Srivastava et al. (10)	Arabia	20	20	46.20 ± 11.08	37 ± 7.56	15/5	15/5	Serum^a^^,^^f^^,^^h^^,^^i^
Babiuch et al. (20)	Poland	20	20	—	—	11/9	9/11	Saliva^b^^,^^c^^,^^f^
Metgud and Bajaj (19)	India	30	30	51.7	48.3	—	—	Serum^b^^,^^f^ & Saliva^b^^,^^f^
Shahi et al. (16)	India	12	45	44.2 ± 11.9	41.7 ± 12.3	—	29/16	Serum^a^^,^^b^^,^^f^^,^^h^
Bose et al. (18)	India	23	23	23–40	23–40	—	—	Serum^d^^,^^e^^,^^f^
Güven et al. (23)	India	9	11	42	34	6/3	20/20	Saliva^b^
Shetty et al. (22)	India	50	65	20–60	20–60	—	—	Saliva^b^
Sachdev et al. (15)	India	70	70	20–60	20–60	—	—	Serum^a^^,^^d^^,^^e^^,^^f^^,^^i^
Ganesan and Kumar (13)	India	10	20	42.1–52.8	42.1–52.8	—	—	Serum^a^^,^^b^ & Saliva^b^
Jain et al. (17)	India	10	10	—	—	—	—	Serum^g^
Kaur et al. (21)	India	40	40	49 ± 5.9	48.9 ± 7.0	20/20	20/20	Serum^d^^,^^e^ & Saliva^b^^,^^c^
Nagao et al. (40)	Japan	38	152	62.4 ± 10.0	61.9 ± 9.9	38/0	152/0	Serum^e^

a SOD.

b MDA.

c 8-OHdG.

d vitamin C.

e Vitamin E.

f GSH.

g CAT.

h catalase.

i GPx.

**Table 2:** Quality score of the included studies.

The first author, publication year	Selection (0–4)	Comparability (0–2)	Exposure (0–3)	Total score
Kuthoor et al. (14)	***	**	***	8
Srivastava et al. (10)	****	*	***	8
Babiuch et al. (20)	****	**	***	9
Metgud and Bajaj (19)	***	**	***	8
Shahi et al. (16)	****	*	***	8
Bose et al. (18)	****	**	***	9
Güven et al. (23)	***	**	***	8
Shetty et al. (22)	***	**	***	8
Sachdev et al. (15)	****	**	***	9
Ganesan and Kumar (13)	****	*	***	8
Jain et al. (17)	***	**	***	8
Kaur et al. (21)	***	**	***	8
Nagao et al. (40)	***	*	***	7

### 3.3 Meta-analysis of oxidative stress markers

#### 3.3.1 Levels of malondialdehyde (MDA)

Three studies (13, 16, 19), including 95 healthy controls and 52 patients with OLK from India, revealed no significant difference in serum MDA levels between patients with OLK (MD: 0.36, 95% CI: −0.04 to 0.76, *p* = 0.08, *I*^2^ = 96%) (Figure 2A). However, six studies (13, 19–23), comprising 186 healthy controls and 159 patients with OLK from India and Poland, showed that the saliva MDA levels were significantly elevated in patients with OLK compared to healthy controls (MD: 0.26, 95% CI: 0.22 to 0.3, *p* < 0.02, *I*^2^ = 63%) (Figure 2B).

### 3.4 Meta-analysis of antioxidant markers

#### 3.4.1 Levels of serum CAT

Four studies (10, 15–17), including 112 patients with OLK and 145 healthy controls from India, indicated that serum CAT levels were significantly decreased in OLK patients compared to healthy controls (MD: -25.71, 95% CI: −40.09 to −11.33, *p* = 0.0005, *I*^2^ = 100%) (Figure 3A).

#### 3.4.2 Levels of serum GPx

Two studies (10, 15), including 90 healthy controls and 90 patients with OLK from Saudi Arabia and India, demonstrated that serum GPx levels were significantly decreased in OLK patients relative to healthy controls (MD: −9.27, 95% CI: −15.72 to −2.82, *p* = 0.005, *I*^2^ = 99%) (Figure 3B).

#### 3.4.3 Levels of serum SOD

Five studies (10, 13–16), consisting of 170 healthy controls and 141 patients with OLK from India, demonstrated that the levels of serum SOD were significantly decreased in the OLK patients compared to the healthy controls (MD: −6.25, 95% CI: −11.45 to −1.05, *p* = 0.02, *I*^2^ = 100%) (Figure 3C).

#### 3.4.4 Levels of serum GSH

Five studies (10, 15, 16, 18, 19), comprising 188 healthy individuals and 155 patients with OLK from Saudi Arabia and India, revealed that the serum GSH levels were significantly decreased in the OLK patients compared to the healthy controls(MD: −8.6, 95% CI: −12.97 to −4.23, *p* < 0.00001, *I*^2^ = 99%) (Figure 3D).

## 4 Discussion

This meta-analysis provides comprehensive evidence characterizing the redox status in patients with OLK. Our findings revealed that OS markers (such as MDA) were significantly elevated in individuals with OLK, while antioxidant stress markers (such as SOD and GPx) were significantly decreased. These results substantiate the hypothesis that OS is a potential key protagonist in the pathological evolution of OLK.

In biological systems, the equilibrium between ROS generation and antioxidant scavenging is crucial for homeostasis (24). A meta-analysis has indicated that antioxidant and OS markers are significant prospective biomarkers for detecting oral lichen planus (OLP), characterized by elevated oxidant levels and diminished antioxidant levels, which may imply the presence of the condition (25). In addition, our previous study also showed that antioxidant and OS markers may be markers of OSF (26). Our analysis aligns with previous findings in OLP and OSF, suggesting a common oxidative etiology across premalignant oral lesions. Despite their differences in pathological characteristics, clinical presentations, and pathophysiology, all three represent aberrant lesions of the oral mucosa that pose a risk of malignant development.

The observed elevation in MDA, a stable by-product of lipid peroxidation (LP), is particularly concerning. ROS provoke LP via interfering with the LP chain reaction, the CYP2E1 enzymatic system, and the antioxidant defense mechanisms (27). LP can lead to the generation of active aldehydes, which subsequently disrupt cell membrane integrity and functionality by inducing inflammation and altering cellular pathways, hence facilitating disease progression (28). The accumulation of MDA in the saliva of OLK patients implies ongoing structural damage to the oral epithelium. This chronic oxidative assault may induce DNA adducts and mutations, thereby fostering a microenvironment conducive to malignant transformation.

Antioxidants are essential for maintaining redox balance and protecting cells from oxidative damage. Both enzymatic and non-enzymatic endogenous antioxidants collaborate to protect tissues and cells from damage induced by free radicals (29). SOD acts as the first line of defense by dismutating superoxide radicals. CAT and GPx are two crucial antioxidant enzymes that alleviate oxidative damage by catalyzing the decomposition of hydrogen peroxide (H_2_O_2_) into water and oxygen (30). GSH is a tripeptide that neutralizes free radicals in the body. Due to its susceptibility to oxidation by specific agents, GSH safeguards the sulfhydryl groups in numerous proteins and enzymes within the body against oxidative damage, hence preserving the appropriate physiological activities of these biomolecules (31). The significant reduction in enzymatic (SOD, CAT, GPx) and non-enzymatic (GSH) antioxidants observed in this study. Decreased enzyme levels in individuals with OLK impair the clearance of ROS, resulting in an accumulation of molecules like H_2_O_2_. This accumulation can induce lipoperoxidation, compromise cell membrane integrity, alter cellular function, and elevate the risk of DNA mutation and malignant transformation (32). While the significant reduction in antioxidant levels observed in this study is evident, the underlying mechanisms driving this decrease warrant further discussion. Emerging evidence suggests that epigenetic modifications can regulate the expression of genes involved in ROS generation and the antioxidant defense system, thereby influencing OS levels and ultimately disease pathogenesis (33). DNA methylation is one of the most common epigenetic modifications, typically occurring at CpG islands within gene promoter regions. This modification often leads to transcriptional silencing and reduced gene expression (34). For instance, specific methylation products of proteins or nucleic acids can directly inhibit the expression of antioxidant or mitochondrial functional genes, leading to ROS production and accumulation (35). Another study systematically reviewed epigenetic data from myocardial tissue in patients with diabetic cardiomyopathy, and demonstrated that hypermethylation of H4K20me3 in the SOD2 promoter region resulted in downregulation of SOD2 expression, leading to elevated intracellular ROS levels and exacerbated OS (34). A cross-sectional study reported that p16INK4a promoter hypermethylation was significantly associated with the risk of disease occurrence (36). Another study further demonstrated that promoter hypermethylation was significantly more frequent in patients with OLK compared to healthy controls (37). Taken together, these findings suggest a plausible hypothesis: promoter hypermethylation of antioxidant genes may contribute to the reduced enzyme levels observed in OLK patients. Such epigenetic silencing would lead to ROS production and accumulation, promote a state of OS, and potentially drive malignant transformation from OLK to oral cancer. However, there is still limited direct evidence linking the methylation of antioxidant genes to the decreased enzymatic activity in OLK. More research is needed in the future to prove this hypothesis.

**Figure 4:**
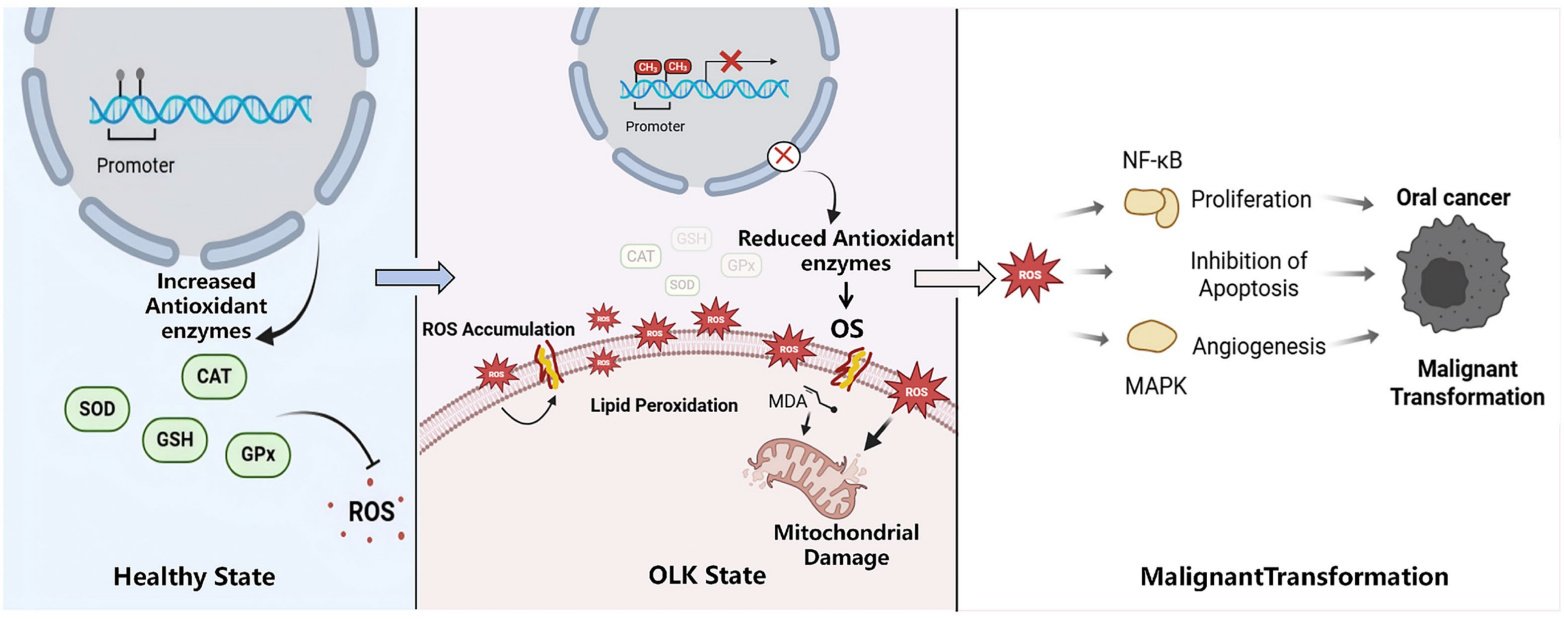
Mechanism of antioxidant enzymes in the development and progression of OLK. In a healthy state, antioxidant enzymes are normally expressed, suppressing the generation of ROS. During the OLK stage, external factors induce abnormal DNA methylation within the cell nucleus. Promoter hypermethylation of antioxidant genes leads to the downregulation of antioxidant enzymes (CAT, GPx, SOD, GSH), resulting in ROS accumulation and the onset of OS. ROS attack mitochondria, causing mitochondrial damage, and simultaneously trigger lipid peroxidation, producing MDA, which further disrupts cellular structure. By activating the NF-κB pathway, ROS drive abnormal cell proliferation, inhibit apoptosis, and promote angiogenesis, ultimately leading to malignant transformation. Created with BioRender.com.

Besides, increased levels of oxidative stress indicators (MDA) and reduced antioxidant markers (SOD, CAT, GSH, GPx) indicate a disrupted redox equilibrium in individuals with OLK, potentially resulting in cellular damage and facilitating malignant transformation. OS has been demonstrated to activate signaling pathways such as NF-κB and MAPK, promote cell proliferation, inhibit apoptosis, and enhance angiogenesis, all of which are essential in tumorigenesis (38, 39). This dysfunctional environment may foster conditions conducive to the development of OLK into malignancy, the mechanism diagram is shown in Figure 4. Consequently, the examination of oxidative stress markers can facilitate the early diagnosis and treatment of illnesses, while antioxidant supplementation or modulation of oxidative stress pathways may aid in reestablishing redox equilibrium, mitigating oxidative damage, and averting the advancement of oral leukoplakia to oral cancer.

The methodological quality of the 13 included case–control studies was assessed using the NOS. The scores ranged from 7 to 9, indicating overall high quality. However, some studies lost points primarily in the Selection domain. Specifically, some studies (14, 19, 22, 23) did not clearly describe the selection of controls, and two studies (21, 40) had potential issues with the representativeness of the cases. Some studies (10, 13, 16, 40) lost points in the comparability domain due to inadequate control for potential confounding factors such as age and sex. This methodological limitation likely contributes to the heterogeneity observed in our meta-analysis, as discussed below.

This study does have some limitations, such as the number of included studies was too small. Moreover, the findings of these studies exhibited significant heterogeneity, which could be attributed to various factors. First, the population included in the study varied in age range and sex proportions. Studies (41, 42) indicates that males possess a higher baseline metabolic rate and generate greater quantities of oxidative metabolites, such as ROS, resulting in elevated oxidative stress levels, hence rendering them more susceptible than females. Furthermore, clinical and experimental studies indicate that females possess a superior antioxidant capacity relative to males, potentially attributable to the antioxidant properties of estrogen, which may render them less susceptible to OS. Thus, differing ratios of males and females may possibly account for the observed heterogeneity. Second, OLK is categorized according to its histological degree of hyperplasia into mild dysplasia, moderate dysplasia, and severe dysplasia, or premalignant lesions. The variability in disease stages among the research populations may contribute to the heterogeneity of the outcomes. Srivastava et al. (10) found that the GSH and GPx levels were markedly reduced in patients with OLK in comparison to the control group. The expression level of GSH diminished progressively with the worsening of pathogenic conditions. Finally, the methods employed for analyzing serum or salivary samples were not uniform. For instance, in one study (19), GSH levels were measured using the method by Srivastava et al. (10) while in another study, GSH was measured using the method developed by IntHout et al. (43) different detection methods may contributions to differences in experimental results. We tried to solve the problem, due to the limited number of literature, subgroup was not possible. Therefore, a random-effects model was applied, although heterogeneity persisted. Additionally, the prediction interval (PI) assists in clinically interpreting the heterogeneity by providing an estimate of the potential treatment effects in future settings. Therefore, we also calculated the PI, and the results showed that the PI values of all results aligned with the CI trends and original MD values. This consistency indicates that in future studies, the same result will be observed, lending credibility to the findings.

## 5 Conclusion

This meta-analysis suggests that OS may be involved in the pathogenesis of OLK. The pooled analysis showed increased levels of the OS marker MDA and decreased levels of antioxidant markers (CAT, GSH, GPx, SOD) in patients with OLK, indicating a potential disruption of redox balance. These alterations could hypothetically contribute to the pathogenesis and potential malignant transformation of OLK, although this remains to be confirmed. Multi-center, large-sample clinical trials should be conducted to enhance the reliability of the results in the future.

## Data Availability

The datasets used and/or analysed during the current study are available from the corresponding author on reasonable request.
