# Supplementary material for: Oxidative stress and antioxidant markers in oral leukoplakia: a systematic review and meta-analysis
Source: Front Med (Lausanne). 2026 Apr 21;13:1807075. doi: 10.3389/fmed.2026.1807075 (PMC13138961; doi:10.3389/fmed.2026.1807075)
Supplement: Supplementary file 1 [file Table_1.DOCX]

**PUBMED**

| #1 | "Leukoplakia, Oral"[MeSH] |
| --- | --- |
| #2 | ((Oral Leukoplakia[Title/Abstract]) OR (Oral Leukokeratosis[Title/Abstract]) OR (Oral Leukokeratoses[Title/Abstract]) OR (Oral Keratoses[Title/Abstract])) |
| #3 | #1 OR #2 |
| #4 | ((((((((((((((((((((((((((((((((((((((oxidative stress[Title/Abstract]) OR (oxidat[Title/Abstract])) OR (antioxidat[Title/Abstract])) OR (antioxidant*[Title/Abstract])) OR (nitrosative stress[Title/Abstract])) OR (nitrative stress[Title/Abstract])) OR (nitro-oxidative stress[Title/Abstract])) OR (free radical*[Title/Abstract])) OR (thiobarbituric acid reactive substances[Title/Abstract])) OR (TBARS[Title/Abstract])) OR (nitric oxide[Title/Abstract])) OR (NO[Title/Abstract])) OR (glutathione disulfide[Title/Abstract])) OR (GSSG[Title/Abstract])) OR (uric acid[Title/Abstract])) OR (zinc[Title/Abstract])) OR (reactive oxygen species[Title/Abstract])) OR (ROS[Title/Abstract])) OR (total antioxidant capacity[Title/Abstract])) OR (TAOC[Title/Abstract])) OR (total oxidant status[Title/Abstract])) OR (TOS[Title/Abstract])) OR (total antioxidant status[Title/Abstract])) OR (TAS[Title/Abstract])) OR (oxidative stress index[Title/Abstract])) OR (OSI[Title/Abstract])) OR (malondialdehyde[Title/Abstract])) OR (MDA[Title/Abstract])) OR (superoxide dismutase[Title/Abstract])) OR (SOD[Title/Abstract])) OR (reactive oxygen metabolites[Title/Abstract])) OR (ROM[Title/Abstract])) OR (glutathione[Title/Abstract])) OR (GSH[Title/Abstract])) OR (glutathione peroxidase[Title/Abstract])) OR (GPx[Title/Abstract])) OR (8-hydroxy-deoxyguanosine[Title/Abstract])) OR (8-OHdG[Title/Abstract])) OR (catalase[Title/Abstract]) |
| #5 | #3 AND #4 |

**Embase**

| #1 | ' Leukoplakia ':ab,ti OR ' Oral Leukoplakia ':ab,ti OR ' Oral Leukokeratosis ':ab,ti OR ' Oral Leukokeratoses ':ab,ti OR Oral Keratoses':ab,ti |
| --- | --- |
| #2 | ‘oxidat ‘:ab,ti OR ‘antioxidat ‘:ab,ti OR ‘oxidant ‘:ab,ti OR ‘antioxidant ‘:ab,ti OR ‘redox ‘:ab,ti OR ‘reactive oxygen species ‘:ab,ti OR ‘ROS ‘:ab,ti OR ‘total antioxidant capacity ‘:ab,ti OR ‘TAOC ‘:ab,ti OR ‘total oxidant status ‘:ab,ti OR ‘TOS ‘:ab,ti OR ‘total antioxidant status ‘:ab,ti OR ‘TAS ‘:ab,ti OR ‘oxidative stress index ‘:ab,ti OR ‘OSI ‘:ab,ti OR ‘nitric oxide ‘:ab,ti OR ‘malondialdehyde ‘:ab,ti OR ‘MDA ‘:ab,ti OR ‘superoxide dismutase ‘:ab,ti OR ‘SOD ‘:ab,ti OR ‘reactive oxygen metabolites ‘:ab,ti OR ‘ROM ‘:ab,ti OR ‘glutathione ‘:ab,ti OR ‘GSH ‘:ab,ti OR ‘glutathione peroxidase ‘:ab,ti OR ‘GPx ‘:ab,ti OR ‘8-hydroxy-deoxyguanosine ‘:ab,ti OR ‘8-OHdG ‘:ab,ti OR ‘catalase ‘:ab,ti |
| #3 | #1 AND #2 |

**Cochrane library**

| #1 | MeSH descriptor: [Leukoplakia, Oral] explode all trees |
| --- | --- |
| #2 | ' Oral Leukoplakia ':ab,ti OR ' Oral Leukokeratosis ':ab,ti OR ' Oral Leukokeratoses ':ab,ti OR Oral Keratoses':ab,ti |
| #3 | #1 or #2 |
| #4 | ‘OSI ‘:ab,ti OR ‘nitric oxide ‘:ab,ti OR ‘malondialdehyde ‘:ab,ti OR ‘MDA ‘:ab,ti OR ‘superoxide dismutase ‘:ab,ti OR ‘SOD ‘:ab,ti OR ‘reactive oxygen metabolites ‘:ab,ti OR ‘ROM ‘:ab,ti OR ‘glutathione ‘:ab,ti OR ‘GSH ‘:ab,ti OR ‘glutathione peroxidase ‘:ab,ti OR ‘GPx ‘:ab,ti OR ‘8-hydroxy-deoxyguanosine ‘:ab,ti OR ‘8-OHdG ‘:ab,ti OR ‘catalase ‘:ab,ti OR ‘antioxidat ‘:ab,ti OR ‘oxidant ‘:ab,ti OR ‘antioxidant ‘:ab,ti |
| #5 | #3 and #4 |

**WOS**

| #1 | TS=‘Leukoplakia) OR AB= ‘Oral Leukoplakia OR Oral Leukokeratosis OR Oral Leukokeratoses OR Oral Keratoses) |
| --- | --- |
| #2 | AB=‘oxidat OR antioxidat OR oxidant OR antioxidant OR redox OR reactive oxygen species OR ROS OR total antioxidant capacity OR TAOC OR total oxidant status OR TOS OR total antioxidant status OR TAS OR oxidative stress index OR OSI OR nitric oxide OR malondialdehyde OR MDA OR superoxide dismutase OR SOD OR reactive oxygen metabolites OR ROM OR glutathione OR GSH OR glutathione peroxidase OR GPx OR 8-hydroxy-deoxyguanosine OR 8-OHdG OR catalase) |
| #3 | ‘TS=‘Leukoplakia) OR AB= ‘Oral Leukoplakia OR Oral Leukokeratosis OR Oral Leukokeratoses OR Oral Keratoses)) AND ‘ AB=‘oxidat OR antioxidat OR oxidant OR antioxidant OR redox OR reactive oxygen species OR ROS OR total antioxidant capacity OR TAOC OR total oxidant status OR TOS OR total antioxidant status OR TAS OR oxidative stress index OR OSI OR nitric oxide OR malondialdehyde OR MDA OR superoxide dismutase OR SOD OR reactive oxygen metabolites OR ROM OR glutathione OR GSH OR glutathione peroxidase OR GPx OR 8-hydroxy-deoxyguanosine OR 8-OHdG OR catalase)) |
